# Clinical Recognition of Frontotemporal Dementia with Right Anterior Temporal Predominance; Recommendations of the International Working Group

**DOI:** 10.1002/alz.092661

**Published:** 2025-01-03

**Authors:** Hulya Ulugut

**Affiliations:** ^1^ Memory & Aging Center, Department of Neurology, University of California in San Francisco, San Francisco, CA USA

## Abstract

**Background:**

Although frontotemporal dementia (FTD) with right anterior temporal lobe (RATL) predominance has been recognized as a separate FTD subtype, a uniform description of the syndrome is still missing. This multicenter study, led by an international working group (IWG), aims to establish a cohesive clinical phenotype and lay the groundwork for consensus on terminology and diagnostic standards.

**Method:**

Retrospective clinical data were systematically collected across 18 dementia centers. The harmonized data were interpreted by 90 FTD experts across over 30 centers. Visual assessments of initial neuroimaging identified study cases, culminating in a cohort of 360 FTD patients with predominant RATL atrophy.

**Result:**

Common symptoms were mental rigidity and preoccupations (78%), disinhibition (74%), naming/word‐finding difficulties (70%), memory deficit (67%), apathy (65%), loss of empathy (65%), and face recognition deficit (60%), based on the chart reviews. Reviewing case notes beyond clinicians' interpretations unveiled impairments in identifying landmarks, smells, sounds, tastes, and bodily sensations, and altered reactions to those stimuli (74%). These symptoms, while not explicitly queried or examined, were often categorized under language, memory, behavioral, psychiatric, or other/unclassified problems by various centers. Available cognitive test scores indicated impairments in emotion, people, social interactions, and visual semantics related domains. Secondly, the IWG interpreted symptoms within three main domains; (i) semantic deficit for predominant non‐verbal information including people, taste, smell, sound, landmarks, bodily sensations, socioemotional information/ concepts/ facts (ii) socioemotional dysfunction including altered emotional expressivity, and emotional apathy, (iii) altered thought process including mental rigidity, hyper‐focused specific interests and altered hedonic valuation and prioritization. Lastly, 67 patients had genetic and/or pathological confirmation for FTLD, and 21 had amyloid positivity. Of note, caution is advised in interpreting amyloid positivity, and the distinct clinical nuances associated with each molecular subtype are emphasized by the IWG.

**Conclusion:**

This collaborative effort represents the first international initiative to establish the largest RATL cohort, resolving contradictions in the field and shedding light on challenges in recognizing RATL impairment in clinical practice. Our ultimate goals are reaching a consensus on diagnostic criteria that will facilitate the recognition of FTD associated with RATL atrophy and differentiate the syndrome from other dementia subtypes and psychiatric disorders.

